# Generation of functional human hepatocytes in vitro: current status and future prospects

**DOI:** 10.1186/s41232-019-0102-4

**Published:** 2019-07-02

**Authors:** Tomoko Yamaguchi, Juntaro Matsuzaki, Takeshi Katsuda, Yoshimasa Saito, Hidetsugu Saito, Takahiro Ochiya

**Affiliations:** 1 Division of Pharmacotherapeutics, Keio University Faculty of Pharmacy, 1-5-30 Shibakoen, Minato-ku, Tokyo, 105-8512 Japan; 2 Division of Molecular and Cellular Medicine, National Cancer Center Research Institute, 5-1-1 Tsukiji, Chuo-ku, Tokyo, 104-0045 Japan; 3 Division of Gastroenterology and Hepatology, Department of Internal Medicine, Keio University School of Medicine, 35 Shinanomachi, Shinjuku-ku, Tokyo, 160-8582 Japan; 4 Institute of Medical Science, Tokyo Medical University, 6-1-1 Shinjuku, Shinjuku-ku, Tokyo, 160-8402 Japan

**Keywords:** Hepatocyte, Regeneration, Progenitor cells, Drug metabolism

## Abstract

Liver and hepatocyte transplantation are the only effective therapies for late-stage liver diseases, in which the liver loses its regenerative capacity. However, there is a shortage of donors. As a potential alternative approach, functional hepatocytes were recently generated from various cell sources. Analysis of drug metabolism in the human liver is important for drug development. Consequently, cells that metabolize drugs similar to human primary hepatocytes are required. This review discusses the current challenges and future perspectives concerning hepatocytes and hepatic progenitor cells that have been reprogrammed from various cell types, focusing on their functions in transplantation models and their ability to metabolize drugs.

## Background

The prognosis of patients with end-stage liver cirrhosis and fulminant hepatitis is poor unless they receive a liver transplant [1]. Unfortunately, there is a shortage of transplantable organs, and consequently, alternatives have been explored. Although the resected human liver has an enormous regenerative capacity [2], the functions of primary human hepatocytes decrease upon conventional two-dimensional culture on an extracellular matrix-coated surface. Functional human hepatocytes can be generated in vitro due to recent technological advances in the stem cell research field [3]. This approach could be an abundant source of cells for therapeutic applications. In addition, in vitro culture of human hepatocytes and/or their progenitors may help to increase understanding of liver development and regeneration following injury, to estimate the risk of drug-induced liver injury, to analyze the interactions between hepatocytes and hepatitis virus, to elucidate the mechanisms underlying liver carcinogenesis, and to assist the development of personalized therapies for patients with hepatocellular carcinoma. This review discusses the current challenges associated with therapeutically relevant approaches for regenerating hepatocytes in vitro and future perspectives for hepatocytes and hepatic progenitor cells reprogrammed from various cell types. Particular focus is paid to the functions of these cells in transplantation models and their ability to metabolize drugs.

## Main text

### Animal models for hepatocyte transplantation experiments

Evaluation of the repopulation rate and hepatic function of transplanted human primary hepatocytes has increased over the past two decades with the development of various mouse models (Table 1). There are three main mouse models: albumin (ALB) uroplasminogen activator (uPA) transgenic mice, mice with knockout of the fumarylacetoacetate hydrolase (Fah) gene, and ALB thymidine kinase transgenic-NOD-SCID-interleukin common mice gamma chain knockout (TK-NOG) mice [19].

**Table 1:** Comparison of potential cell sources for cell-based treatment of liver failure

	Cell source	Methods	Features	CYP positivity and activity in vitro	Animal model	Repopulation efficiency	Serum human ALB concentration	Ref.
DE cells	iPSCs	Activin A	Prolonged survival	–	NSG mice treated with DMN (chronic liver injury)	13–35%	–	[4]
ICG^high^ HL cells	ESCs	Lithium, OSM, DEX, and HGF	Gamma-glutamyl transpeptidase activity, glycogen accumulation, and urea secretion	Expression of CYP1A2 and CYP3A4	BALB/c nude mice treated with CCl_4_ (acute liver injury)	10.2 ± 3.11% at 7 weeks	3 μg/ml at 7 weeks	[5]
iPSC-LBs	iPSCs	Coculture with endothelial and mesenchymal cells	Early hepatic marker positive and connections between human and host vessels	Expression of CYP3A7	TK-NOG mice	–	1.7 μg/ml at 6 weeks	[6]
HL cells	ESCs/iPSCs	Activin A, FGF, HGF, and DEX	LDL uptake, lipid storage, glycogen storage, and uptake and excretion of ICG	Expression of CYP3A4	*MUP-uPA*/SCID/Bg mice	1–20% at 100 days	0.1–6.4 mg/ml at 100 days	[7]
iMPC-Heps	Fibroblasts	OCT4, SOX2, KLF4, CHIR99021, DLPC, NaB, Par, RG, Activin A, bFGF, EGF, A83-01, BMP4, DEX, HGF, OSM, and Compound E	Hepatocyte marker positive	Activities of CYP3A4 and CYP2C19	FRG mice	2% at 9 months	100 μg/ml at ~ 35 weeks	[8]
iHeps	Fibroblasts	HNF6, HNF4α, HNF1α, CEBPA, PROX1, and ATF5	Glycogen synthesis, LDL uptake, exclusion of absorbed ICG, and accumulation of fatty droplets	Activities of CYP3A4, CYP1A2, and CYP2B6	Tet-uPA/Rag2^−/−^/γc^−/−^ mice	30% at 7 weeks	150 μg/mL at 7 weeks	[9]
iHeps/iHeps^LT^	Fibroblasts	FOXA3, HNF1β, and HNF4α (SV40)	Hepatocyte marker positive, glycogen storage, ICG absorption, acetylated LDL uptake, and cytoplasmic accumulation of triglycerides and lipids	Activities of CYP1A2, CYP2A, and CYP2D6	F/R mice	0.3–4.2% at 9 weeks	350 ng/ml at 9 weeks	[10]
hiEndoPC-Heps	Gastric epithelial cells	Bay, Bix, RG, SB, BMP4, Wnt3a, FGF4, HGF, DEX, and OSM	Uptake of ICG and LDL, glycogen storage, and accumulation of fatty droplets	CYP3A4 activity	F/R mice	10% at 8–10 weeks	350 ng/ml at 8 weeks	[11]
CFPHs	Hepatocytes	Long-term culture	Liver progenitor cell marker positive	Expression of CYP2C9, CYP2C19, CYP1A1, and CYP1A2	uPA/SCID mice	0.2–27.0% at 9–10 weeks	9–728 μg/mL at 9–10 weeks	[12, 13]
Human liver organoid	Ductal cells (EPCAM+)	N2, B27, *N*-acetylcysteine, gastrin, EGF, R-spondin1, FGF10, HGF, nicotinamide, A83-01, and FSK	Hepatocyte morphology, glycogen accumulation, and LDL uptake	CYP3A4 activity	BALB/c nude mice treated with CCl4 (acute liver injury)	–	80 ng/ml at 6 weeks	[14]
hCdHs	Hepatocytes	HGF, A83-01, and CHIR99021	High nucleus-to-cytoplasm ratio, pluripotency stem cell marker positive, and hepatic progenitor cell marker expression	CYP1A2 activity	Alb-TRECK/SCID mice	–	1.5 μg/ml	[15]
HepLPCs	Hepatocytes	N2, B27, HGF, EGF, Y27632, A83-01, CHIR99021, S1P, and LPA	High nucleus-to-cytoplasm ratio and liver progenitor cell marker expression	Activities of CYP1A2, CYP2B6, and CYP3A4	FRG mice	13%	–	[16]
ProliHHs	Hepatocytes	Wnt3a, N2, B27, *N*-acetylcysteine, gastrin, EGF, FGF10, HGF, nicotinamide, A83-01, FSK, and Y27632	Progenitor-associated gene expression and biphenotypic cells	CYP2B6 activity	FRG mice	64 ± 21.8% (at P4) at 4 months	5.8 ± 4.5 mg/ml (at P4) at 4 months	[17]
Hep-Orgs	Fetal and cryopreserved primary hepatocytes	RSPO1 conditioned medium, B27, EGF, *N*-acetylcysteine, gastrin, CHIR99021, HGF, FGF7, FGF10, A83-01, nicotinamide, Rho inhibitor g-27,632, and TGFα	Networks of bile canaliculi, PAS staining, and LDL uptake	CYP3A4 activity and CYP2E1 expression	FNRG mice	–	200 μg/ml after 90 days	[18]

In uPA/SCID mice, constitutive expression of uPA in hepatocytes causes liver injury and permits selective expansion of transplanted human hepatocytes. However, uPA/SCID mice have some disadvantages. Repopulation of human hepatocytes in the liver of these mice is decreased due to deletion of the uPA transgene by homologous recombination. In addition, hemizygotes cannot be used as hosts because homologous recombination occurs more frequently in hemizygotes than in homozygotes. To overcome these disadvantages, Tateno et al. established a novel host strain that expresses a transgene comprising the ALB promoter/enhancer and uPA cDNA and is of a SCID background (cDNA-uPA/SCID mice) [20]. Tesfaye et al. also generated a novel mouse strain that expresses the uPA gene under the control of the major urinary protein promoter and is of a SCID/beige background (MUP-uPA/SCID/Bg mice) [21]. cDNA-uPA/SCID mice have the following advantages: their body is larger than that of uPA/SCID mice, it is easier to perform animal experiments, and the frequency of renal damage is decreased. MUP-uPA/SCID/Bg mice provide a long time window (up to 12 months) for hepatocyte engraftment and are efficiently infected with hepatitis B virus or hepatitis C virus [22]. Tet-uPA/Rag2^−/−^/γc^−/−^ mice are easily bred, remain healthy prior to induction of liver injury, and have no time-window limit for liver cell transplantation.

In Fah-knockout mice, deletion of Fah, which functions in the tyrosine catabolic pathway, causes accumulation of toxic fumarylacetoacetate, resulting in liver injury. Liver disease can be controlled by administering 2-(2-nitro-4-trifluoromethylbenzoyl)-1,3-cyclohexanedione in these mice. Azuma et al. generated Fah^−/−^/Rag2^−/−^/Il2rg^−/−^ (FRG) mice by crossing Fah-knockout mice and Rag2^−/−^/Il2rg^−/−^ mice, which are immunodeficient and lack B, T, and NK cells [23]. The capacity for liver xeno-repopulation is reduced in Fah^−/−^Rag2^−/−^ (F/R) mice due to the presence of NK cells [24]. However, F/R mice are easy to bred and tolerate hepatocyte transplantation. Fah^−/−^ NOD Rag1^−/−^Il2rg^−/−^ (FNRG) mice are more immunodeficient than FRG mice [25].

A herpes simplex virus type 1 thymidine kinase (HSVtk) transgene was expressed in the liver of highly immunodeficient NOG mice. Ganciclovir can control the hepatotoxic transgene in TK-NOG mice. In addition, TK-NOG mice mimic liver zonation and drug metabolism in the repopulated liver [26].

Azuma et al. intrasplenically transplanted human hepatocytes into FRG mice [23]. Human hepatocytes repopulated the livers of these mice with a repopulation rate of > 80%. Hasegawa et al. intrasplenically transplanted human liver cells into TK-NOG mice [26]. The repopulation rate was 43% in the livers of these mice. Tateno et al. intrasplenically transplanted human hepatocytes into cDNA-uPA/SCID mice [20]. The repopulation rate was > 70% in the livers of these mice. Thus, transplanted mature human hepatocytes demonstrate a high capacity to regenerate the injured liver in mice, which indicates the feasibility of mouse models for checking the function of in vitro-derived cells.

### Potential alternative cell sources for hepatocyte transplantation therapy

To overcome the shortage of donor hepatocytes, many attempts have been made to generate functional hepatocytes from multiple types of cells (Table 1). However, there is controversy regarding the usefulness of these cells for transplantation therapy. Liu et al. generated human induced pluripotent stem cell (iPSC) lines from different sources and intravenously transplanted definitive endoderm (DE) cells differentiated from these iPSC lines into *NOD/Lt-SCID/IL-2Rγ*^*−/−*^ (NSG) mice that had been treated with dimethylnitrosamine (DMN) for 4 weeks (liver cirrhosis model) [4, 27–30]. The engraftment percentage, calculated as the percentage of human hepatic cells expressing ALB, was 13% in the livers of mice transplanted with 2 × 10^6^ DE cells and 35% in the livers of mice transplanted with 7 × 10^6^ DE cells. Woo et al. reported that embryonic stem cells (ESCs) treated with lithium and cultured in the presence of hepatocyte growth factor (HGF), oncostatin M (OSM), and dexamethasone (DEX) differentiated into cells with a hepatocyte-like (HL) morphology that expressed ALB and keratin 18, and that HL cells with high liver function were enriched using indocyanine green (ICG) [5, 31–34]. When HL ICG^high^ cells were transplanted into CCl_4_-intoxicated BALB/c mice (acute liver injury model), the percentage of human ALB-positive cells was lower at day 35 (10.2 ± 3.11%) than at day 3 (20.2 ± 4.45%) after transplantation. Takebe et al. revealed that hepatic endoderm cells derived from human iPSCs formed a three-dimensional spherical tissue mass termed iPSC-derived liver buds (iPSC-LBs), which expressed early hepatic marker genes, upon culture with human umbilical vein endothelial cells and human mesenchymal stem cells [6]. In vitro-derived human iPSC-LBs integrated with the host vasculature within 48 h after transplantation. Human iPSC-LBs began producing ALB at approximately day 10 post-transplantation in TK-NOG mice and increased the concentration of ALB to 1.983 μg/ml by day 45. Carpentier et al. demonstrated that HL cells differentiated from iPSCs via a multistep protocol were positive for α-1-antitrypsin (AAT) and Forkhead box a2 (FOXA2), which are endoderm cell markers, as well as hepatocyte nuclear factor 4 alpha (HNF4α), which is a master regulator of hepatic differentiation. Upon transplantation of HL cells into the spleen of *MUP-uPA*/SCID/Bg mice, the human ALB concentration at day 10 post-engraftment was 50–3900 μg/ml [7, 35, 36].

Transdifferentiation, which refers to direct conversion of a differentiated cell type into another one without an intermediary pluripotent stage, could be an alternative to iPSCs for generation of functional hepatocytes. Zhu et al. transduced human fibroblasts with retroviruses expressing OCT4, SOX2, and KLF4 and then replated these cells into a medium containing established growth factors and CHIR99021 (a GSK-3β inhibitor) for reprogramming into endoderm cells [8]. Upon addition of A83-01 (a transforming growth factor-β inhibitor) and Compound E (a Notch signaling inhibitor) to inhibit biliary differentiation, these cells differentiated into induced multipotent progenitor cell hepatocytes (iMPC-Heps) that expressed hepatocyte markers. Following transplantation of iMPC-Heps into FRG mice, human ALB was detected in mouse serum at 2 months post-transplantation and reached a concentration of 104 μg/ml after 6 months, with a liver repopulation efficiency of 2%. Du et al. demonstrated that overexpression of HNF6, HNF4α, and HNF1α induced differentiation of fibroblasts into cells that were morphologically similar to hepatocytes (3H cells). They also overexpressed CEBPA, PROX1, and ATF5 in 3H cells and observed a dramatic morphological change of fibroblasts into epithelial cells within 1 week (iHeps) [9]. iHeps were intrasplenically transplanted into Tet-uPA/Rag2^−/−^/γc^−/−^ mice [37]. The concentration of human ALB in mouse serum gradually increased and peaked at 313 ng/ml at 7 weeks post-transplantation, with a repopulation efficiency of approximately 30%. Huang et al. reported that overexpression of FOXA3, HNF1β, and HNF4α induced high levels of hepatic gene expression in fibroblasts at 12 days after induction (iHeps) [10]. When iHeps transfected with the SV40 large T antigen were transplanted into F/R mice, staining of human Fah and AAT showed that these cells repopulated 0.3–4.2% of the liver parenchyma in surviving mice [23]. Transdifferentiation of fibroblasts was induced via gene transfer in these three reports. On the other hand, Wang et al. demonstrated that treatment with four small molecules (Bay K 8644, Bix01294, RG108, and SB431542) converted gastric epithelial cells into induced endodermal progenitor cells (hiEndoPCs) with a multilineage differentiation capacity [11]. Transplanted hiEndoPC-derived hepatic cells (hiEndoPC-Heps) with hepatocyte-specific functions rescued liver failure in F/R mice. Moreover, human ALB levels were comparable to those from either hESC-Heps, with a maximum repopulation efficiency of 10%.

Several recent studies proposed that hepatocytes are a source of expandable hepatic cells. In 2008, Utoh et al. identified a small population of replicative hepatocytes, termed colony-forming parenchymal hepatocytes (CFPHs), in long-term cultures of human adult hepatocytes. The frequency of these cells was 0.01–0.09% depending on donor age [12, 13]. When CFPHs were transplanted into uPA/SCID mice, they engrafted into the liver and grew for at least 10 weeks. Moreover, the maximum repopulation rate was 27% and the maximum human ALB concentration was 728 μg/ml. In an attempt to generate cells that proliferate more rapidly than CFPHs and that exhibit a repopulative capacity and hepatocyte functions after transplantation, we previously reported that a cocktail of three small chemicals, namely, Y27632, A83-01, and CHIR99021 (YAC), effectively converted rodent mature hepatocytes into liver progenitors, termed chemically induced liver progenitors [38]. However, Kim et al. reported that YAC-treated human hepatocytes rapidly died without proliferating [15]. To overcome this problem, they searched for additional hepatic factors that increased the efficiency of conversion. Given that HGF is important for liver organogenesis, liver regeneration, and maintenance of hepatic progenitor cells [39–41], they supplemented the reprogramming medium with this molecule and tested the effects of various combinations of small molecules together with HGF. A combination of HGF and two small molecules, namely, A83-01 and CHIR99021, was most effective. Human chemically derived hepatic progenitors (hCdHs) formed within 10–15 days of treatment with this combination. When hCdHs were transplanted into Alb-TRECK/SCID mice, they engrafted and repopulated about 20% of the diseased parenchyma within 3 weeks, and the ALB concentration reached > 1 μg/ml. Fu et al. developed transition and expansion medium (EM), which can be used to convert human hepatocytes into hepatocyte-derived liver progenitor-like cells (HepLPCs) in vitro [16, 42]. When HepLPC-derived hepatocytes (HepLPC-Heps) were transplanted into F/R mice, human ALB-positive cells covered 7.2–16.1% of the liver parenchyma in surviving mice. In 2015, Huch et al. reported that leucine-rich orphan G-protein-coupled receptor 5-positive cells isolated from the human liver expanded and became bile duct-derived bipotent progenitor cells upon culture in EM [14, 43]. When these cells were engrafted into BALB/c nude mice that had been administered CCl_4_-retrorsine to induce acute liver failure, human ALB was detected in mouse serum within 7–14 days. Using a similar method as culture in the presence of YAC and EM, Zhang et al. revealed that culture in human liver isolation medium, which contained the same supplements as EM and lacked R-spondin1, Noggin, and forskolin, was optimal to generate proliferating human hepatocytes (ProliHHs) and that Wnt3a was the key factor in this medium [17]. This indicates that Wnt3a is more important than CHIR99021 and R-spondin1 in this context. Following transplantation of ProliHHs, 11 of 14 FRG mice survived for more than 4 months, whereas all FRG mice not transplanted with hepatocytes died within 4 months. Importantly, the concentration of human ALB in mouse serum was 5.8 mg/ml after 4 months. The repopulated ProliHHs expressed phase I and II enzymes and transporters at levels comparable with those in primary human hepatocytes after transplantation.

Hu et al. established human fetal hepatocyte organoids with a typical grape-like structure [18]. They also established organoids from cryopreserved primary human hepatocytes, which had small lumina and contained large cells with a hepatocyte morphology. Notably, ALB secretion by the latter organoids was comparable with that by primary human hepatocytes. Organoids were transplanted like hepatocyte transplantations into FNRG mice via splenic injection [44, 45]. At 90 days after transplantation, the serum human ALB in mice transplanted with human fetal hepatocyte organoids had increased by 200-fold to more than 200 μg/ml on average. Fu et al. revealed that three-dimensional spheroid formation enhanced hepatic differentiation in vitro [16]. Zhang et al. reported that ProliHHs matured in three-dimensional organoid culture [17]. Thus, three-dimensional culture may contribute to the maturation of hepatocytes.

### Potential application of in vitro-generated hepatic cells for drug development studies

Primary human hepatocytes are the gold standard for drug development studies. Olson et al. compared drug toxicities between humans and various animals, including dogs, primates, rats, mice, and guinea pigs [46]. Their analysis indicated that the overall concordance between human and animal toxicity was 71%. Many in vitro models of the liver have been used, including liver slices, hepatic cell lines, and primary hepatocytes. Liver tissue slices exhibit zone-specific cytochrome p450 (CYP) activity and phase II enzyme expression; however, these are unstable [47]. Although hepatic cell lines provide an unlimited number of cells, their expression levels of phase I and II enzymes decrease upon repeated passage [48]. Consequently, human hepatocytes that can metabolize drugs and toxicity screening platforms are required. However, the use of primary human hepatocytes is hampered by the limited number of donors and the small number of cells that are obtained. In addition, it is difficult to maintain the proliferative capacity and function of hepatocytes in vitro [49].

Stem cell-derived hepatocytes reportedly exhibit substantial CYP enzyme activity; however, their applicability for drug testing remains controversial. Liu et al. demonstrated that human iPSC-derived hepatocytes exhibited activities of major CYP enzymes, such as CYP1A2, CYP2C9, CYP2C19, and CYP2D6, similar to primary hepatocytes [4]. Woo et al. reported that ICG^high^ HL cells were positive for ALB, keratin 18, HNF4α, and CYP1A2 and that expression of enzymes related to phase I and II drug metabolism, namely, CYP3A4 and glutathione S-transferase 1/2, was enhanced in these cells according to quantitative PCR [30]. Carpentier et al. demonstrated that HL cells exhibited various hepatocyte-specific functions, including uptake of low-density lipoprotein (LDL), storage of lipids based on Oil Red O staining, storage of glycogen based on periodic acid-Schiff staining, and uptake and excretion of ICG; however, HL cells were mainly negative for CYP2D6 and only a few cells were weakly positive for CYP3A4 [32]. These studies collectively suggest that stem cell-derived hepatic cells are useful for pharmaceutical studies. However, they did not demonstrate the inducibility of CYP enzyme activities, which is a major criterion for application of cultured hepatic cells in drug development studies. A few groups described CYP inducibility in terms of enzymatic activity [50–52]. However, the number of such studies is very small, and consequently, the usefulness of stem cell-derived hepatocytes for pharmaceutical studies remains controversial.

Hepatocyte-derived expandable hepatic cells could be used instead of primary human hepatocytes in pharmaceutical studies. Kim et al. reported that omeprazole treatment significantly increased CYP1A2 activity in hCdH-derived hepatocytes relative to that in hCdHs to a similar level as that in primary human hepatocytes [15]. Fu et al. demonstrated that omeprazole treatment increased CYP1A2 expression by 80 ± 11-fold to 193 ± 27-fold, CITCO treatment increased CYP2B6 expression by 10 ± 2-fold to 26 ± 4-fold, and rifampicin treatment increased CYP3A4 expression by 47 ± 2-fold to 96 ± 5-fold (in comparison with the DMSO-treated control) in HepLPCs-Heps [16]. Furthermore, HepLPCs-Heps metabolized acetaminophen, OH-bupropion, OH-diclofenac, OH-testosterone, and OH-coumarin Glu to a similar extent as primary hepatocytes. Zhang et al. reported that CYP2B6 metabolic activity in ProliHHs increased after maturation, in accordance with increased mRNA expression of genes involved in CYP2B6 metabolism [17]. These reports strongly suggest that hepatocyte-derived expandable cells have an advantage over stem cell-derived hepatic cells in terms of CYP inducibility.

### Future perspectives

In the past decade, significant progress has been made in the development of hepatocyte replacement therapy as an alternative to liver transplantation for severe liver failure. Importantly, the use of autologous cell sources would obviate the need for systemic immune suppression, which is required after liver transplantation. Previous reports tend to only describe the ideal data (publication bias), and consequently, it is difficult to compare their results. Approaches to standardize the methods for functional evaluation of these cells must be discussed. Cells must be sufficiently expandable for therapeutic applications. Repeated passage can change the quality of cells. Serum human ALB levels and repopulation efficiencies in several animal models of liver disease provide reliable data to evaluate cell functions. Secretion of ALB by transplanted cells is higher in recent studies than in older studies (Table 1). The safety of cell replacement therapy must also be considered. In particular, the risk of tumor formation following transplantation of cells reprogrammed via gene transfer must be thoroughly investigated. Generation of mature hepatocyte-derived progenitors via treatment with small molecules is currently the best strategy in terms of cell function and safety. Further studies are required to determine whether mature hepatocytes obtained from patients with severe liver disease such as cirrhosis can be converted into progenitors with sufficient functions.

In vitro culture of functional hepatocytes may facilitate the evaluation of drug metabolism, which would accelerate the safety assessment of new drugs. Personalized assessment of the hepatic side effects of drugs may also be possible using in vitro models generated using a person’s own hepatocytes. Therefore, in vitro drug metabolism should be considered when selecting a strategy to generate hepatocytes.

The rapid development of genome editing technologies means that genetic changes can be introduced into hepatocyte progenitors in a site-specific manner, including correction of disease-causing gene mutations in patient-derived hepatocytes. This approach may enable us to cure congenital/inherited metabolic diseases. On the other hand, the introduction of specific mutations into non-diseased hepatocyte progenitors could be used to generate ideal disease models. This approach could be used to investigate the mechanisms underlying liver carcinogenesis.

## Conclusion

**Fig. 1:**
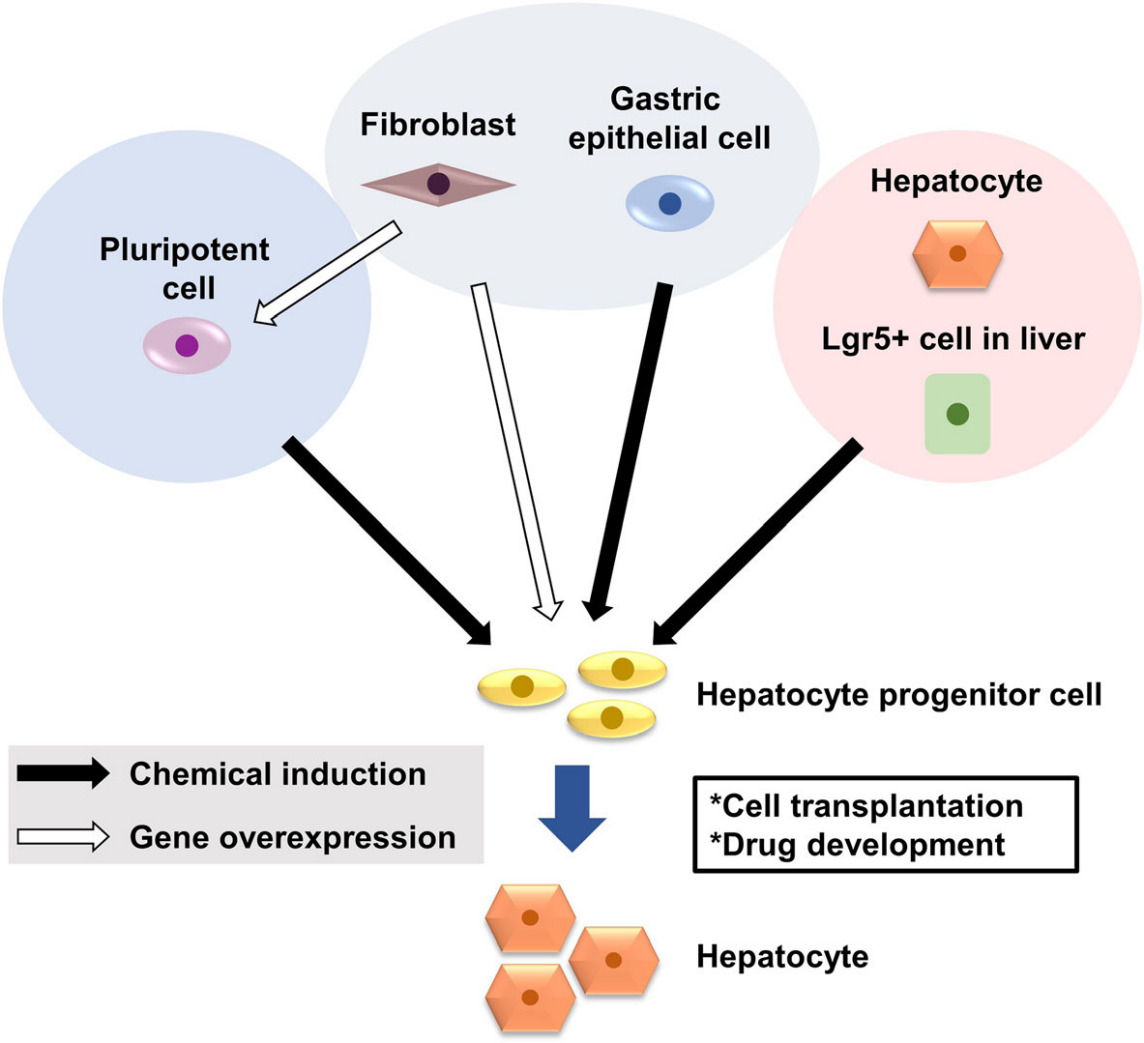
Approaches to generate hepatocyte progenitors in vitro. Current approaches to generate in vitro-expandable hepatocytes include differentiation of human pluripotent stem cells, reprogramming of fibroblasts and cells of a similar developmental origin, identification of liver progenitor cells, and reprogramming of mature hepatocytes. In vitro-expandable hepatocytes are required as a therapeutic alternative to liver transplantation and for drug development

In vitro-expandable hepatocytes are required as therapeutic alternatives to liver transplantation and for drug development. Three strategies have been proposed to generate functional hepatocytes: (i) generation of hepatocytes from ESCs or iPSCs, (ii) transdifferentiation of fibroblasts and other differentiated cells into hepatocytes, and (iii) chemical induction of hepatocyte progenitors from mature hepatocytes (Fig. 1). Standardized methods to evaluate cell functions are required to compare these methods. The coming decade will reveal which strategy holds the most promise for translation into clinical applications.

## Data Availability

Not applicable

## Abbreviations

ALB: Albumin
CFPH: Colony-forming parenchymal hepatocyte
CYP: Cytochrome p450
DE: Definitive endoderm
DEX: Dexamethasone
DMN: Dimethylnitrosamine
EM: Expansion medium
ESC: Embryonic stem cell
F/R: Fah^−/−^Rag2^−/−^
FOXA2: Forkhead box a2
FRG: Fah^−/−^/Rag2^−/−^/Il2rg^−/−^
hCdH: Human chemically derived hepatic progenitor
HepLPC: Hepatocyte-derived liver progenitor-like cell
HepLPC-Hep: HepLPC-derived hepatocyte
HGF: Hepatocyte growth factor
hiEndoPC: Human induced endodermal progenitor cell
hiEndoPC-Hep: hiEndoPC-derived hepatic cell
HL: Hepatocyte-like
HNF4α: Hepatocyte nuclear factor 4 alpha
ICG: Indocyanine green
iMPC-Hep: Induced multipotent cell progenitor hepatocyte
iPSC: Induced pluripotent stem cell
iPSC-LB: iPSC-derived liver bud
LDL: Low-density lipoprotein
NSG: *NOD/Lt-SCID/IL-2Rγ* ^*−/−*^
OSM: Oncostatin M
ProliHH: Proliferating human hepatocyte
YAC: Y27632, A83-01, and CHIR99021
